# Strontium disodium hexa­thio­diphosphate(IV) octa­hydrate

**DOI:** 10.1107/S1600536810025316

**Published:** 2010-07-03

**Authors:** Claus Ehrhardt, Mimoza Gjikaj

**Affiliations:** a Institute of Inorganic and Analytical Chemistry, Clausthal University of Technology, Paul-Ernst-Strasse 4, 38678 Clausthal-Zellerfeld, Germany

## Abstract

The crystal structure of SrNa_2_(P_2_S_6_)·8H_2_O is isotypic with that of its calcium analogue. The asymmetric unit consists of one Sr^2+^ cation (2 symmetry), two Na^+^ cations (2 and 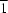 symmetry, respectively), one-half of a centrosymmetric (P_2_S_6_)^4−^ anion with a staggered confirmation and four water mol­ecules. The crystal structure is built up from layers of cations and anions extending parallel to (101). Each SrO_8_ polyhedron is connected *via* edge-sharing to two NaO_4_S_2_ octa­hedra and to one NaO_2_S_4_ octa­hedron. The NaO_4_S_2_ octa­edra are, in turn, connected with two (P_2_S_6_)^4−^ anions through common corners. Adjacent layers are held together by several O—H⋯S hydrogen-bonding inter­actions.

## Related literature

For background to thio­diphosphates(IV), including their crystal structures, see: Jörgens *et al.* (2003 ▶); Klingen *et al.* (1973 ▶). For the synthesis of Na_4_(P_2_S_6_)·6H_2_O, see: Fincher *et al.* (1998 ▶). For the isotypic structure of CaNa_2_(P_2_S_6_)·8H_2_O, see: Ehrhardt &Gjikaj  (2010 ▶).

## Experimental

### 

#### Crystal data

SrNa_2_(P_2_S_6_)·8H_2_O
*M* *_r_* = 532.03
Monoclinic, 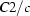

*a* = 14.9010 (19) Å
*b* = 9.3282 (7) Å
*c* = 14.1338 (19) Å
β = 114.918 (10)°
*V* = 1781.7 (4) Å^3^
*Z* = 4
Mo *K*α radiation
μ = 3.98 mm^−1^
*T* = 223 K
0.28 × 0.26 × 0.25 mm

#### Data collection

Stoe IPDS 2 diffractometer
14472 measured reflections
2544 independent reflections
2302 reflections with *I* > 2σ(*I*)
*R*_int_ = 0.064

#### Refinement

*R*[*F* ^2^ > 2σ(*F* ^2^)] = 0.036
*wR*(*F* ^2^) = 0.087
*S* = 1.12
2544 reflections
121 parameters
All H-atom parameters refined
Δρ_max_ = 1.14 e Å^−3^
Δρ_min_ = −0.89 e Å^−3^

### 

Data collection: *X-AREA* (Stoe & Cie, 2008 ▶); cell refinement: *X-AREA*; data reduction: *X-AREA*; program(s) used to solve structure: *SHELXS97* (Sheldrick, 2008 ▶); program(s) used to refine structure: *SHELXL97* (Sheldrick, 2008 ▶); molecular graphics: *DIAMOND* (Brandenburg, 2004 ▶); software used to prepare material for publication: *SHELXL97*.

## Supplementary Material

Crystal structure: contains datablocks I, global. DOI: 10.1107/S1600536810025316/wm2365sup1.cif

Structure factors: contains datablocks I. DOI: 10.1107/S1600536810025316/wm2365Isup2.hkl

Additional supplementary materials:  crystallographic information; 3D view; checkCIF report

## Figures and Tables

**Table 1:** Selected bond lengths (Å)

Sr—O1	2.573 (2)
Sr—O2	2.596 (2)
Sr—O3	2.631 (2)
Sr—O4	2.6459 (19)
Na1—O3	2.345 (2)
Na1—O4	2.372 (2)
Na1—S2^i^	2.9741 (7)
Na2—O2	2.570 (3)
Na2—S1^i^	2.9525 (15)
Na2—S3	2.9924 (9)
P—S1	2.0162 (9)
P—S2	2.0243 (9)
P—S3	2.0248 (9)
P—P^i^	2.2405 (12)

Symmetry code: (i) 
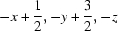
.

**Table 2:** Hydrogen-bond geometry (Å, °)

*D*—H⋯*A*	*D*—H	H⋯*A*	*D*⋯*A*	*D*—H⋯*A*
O1—H1*A*⋯S3^ii^	0.76 (7)	2.66 (6)	3.324 (3)	146 (6)
O1—H1*B*⋯S2^iii^	0.86 (6)	2.53 (6)	3.306 (3)	151 (5)
O2—H2*A*⋯S2^ii^	0.82 (6)	2.51 (6)	3.334 (2)	176 (6)
O2—H2*B*⋯S2^i^	0.79 (5)	2.43 (5)	3.214 (2)	176 (5)
O3—H3*A*⋯S1^iv^	0.76 (7)	2.44 (7)	3.169 (2)	163 (5)
O3—H3*B*⋯S1^ii^	0.88 (7)	2.40 (7)	3.222 (2)	157 (6)
O4—H4*A*⋯S3^v^	0.95 (5)	2.29 (5)	3.245 (2)	175 (4)
O4—H4*B*⋯S3	0.91 (6)	2.30 (6)	3.199 (2)	171 (5)

Symmetry codes: (i) 
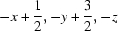
; (ii) 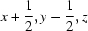; (iii) 
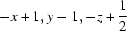
; (iv) 
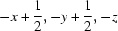
; (v) 
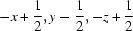
.

**Fig. 1.**
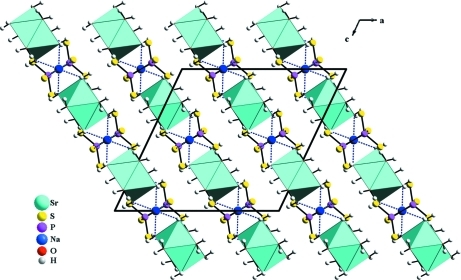
The crystal structure of CaNa2(P2S6).8H2O in a projection along [010].

**Fig. 2.**
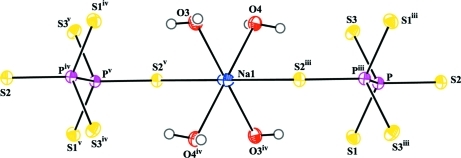
Coordination of Na1 with the atom labelling scheme. The displacement ellipsoids are drawn at the 50% probability level. Symmetry codes as in Table 1. H atoms are represented as spheres of arbirtary radius.

**Fig. 3.**
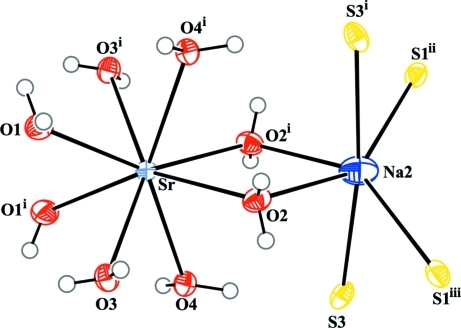
View of the edge-shared CaO8 and Na(2)O2S4 polyhedra with the atom labelling scheme. The displacement ellipsoids are drawn at the 50% probability level. Symmetry codes as in Table 1. H atoms are represented as spheres of arbirtary radius.

## supplementary crystallographic information

### Comment

Alkaline earth hypothiodiphosphates were first reported by Klingen *et al.* (1973). The structure of the title compound, SrNa_2_(P_2_S_6_).8H_2_O, is isotypic with that of its calcium analogue, CaNa_2_(P_2_S_6_).8H_2_O (Ehrhardt & Gjikaj, 2010). The asymmetric unit of SrNa_2_(P_2_S_6_).8H_2_O contains one Sr^2+^ cation, two Na^+^ cations, one half of a (P_2_S_6_)^4-^ anion and four water molecules (Fig. 1)

Na(1) is octahedrally coordinated by four H_2_O molecules and two sulfur atoms of two (P_2_S_6_)^4-^ anions (Fig. 2). Na(2) is also octahedrally coordinated by two H_2_O molecules and four sulfur atoms of two (P_2_S_6_)^4-^ anions (Fig. 3). The strontium cation is eightfold coordinated by water O atoms with Sr—O distances from 2.573 (2) to 2.6459 (19) Å. The SrO_8_ coordination polyhedron can be described as a bicapped trigonal prism.

The crystal structure is built up from layers of cations and anions extending parallel to (101). Within the layer each SrO_8_ polyhedron is connected by edge-sharing to two Na(1)O_4_S_2_ octahedra and to one Na(2)O_2_S_4_ octaedron. Furthermore, the Na(1)O_4_S_2_ octaedra are connected through common corners with two (P_2_S_6_)^4-^ anions.

The discrete ethane-like (P_2_S_6_)^4-^ anion has a staggered conformation and is located on a centre of inversion associated with the midpoint of the P—P bond. The corresponding P—P distance is 2.2405 (12) Å; the P—S distances range from 2.0162 (9) to 2.0248 (9) Å. These values agree well with those reported previously for other hypothiodiphosphate structures (Jörgens *et al.*, 2003).

Neighbouring layers are held together by various O—H···S hydrogen bonding interactions. The donor—acceptor distances between O atoms of water molecules and S atoms of (P_2_S_6_)^4-^ units range from 3.169 to 3.334 Å (Table 2).

With the exception of the *M*-O bond lengths (*M* = Ca, Sr), all other bond lengths and angles as well as the O—H···S hydrogen bonding scheme are very similar in the two isotypic *M*Na_2_(P_2_S_6_).8H_2_O structures.

### Experimental

Na_4_(P_2_S_6_).6H_2_O has been prepared according to Fincher *et al.* (1998). The title compound was obtained by adding a molar equivalent of strontium hydroxide to a solution of Na_4_(P_2_S_6_).6H_2_O in 70 ml distilled water at 348 K. Slow cooling to room temperature yielded colorless crystals of the title compound within some days.

### Refinement

Hydrogen atoms were found from the difference Fourier map and were refined independently from their respective oxygen atoms with individual isotropic displacement parameters.

### Crystal data

**Table:**

SrNa_2_(P_2_S_6_)·8H_2_O	*F*(000) = 1064
*M**_r_* = 532.03	*D*_x_ = 1.983 Mg m^−^^3^
Monoclinic, *C*2/*c*	Mo *K*α radiation, λ = 0.71073 Å
Hall symbol: -C 2yc	Cell parameters from 14971 reflections
*a* = 14.9010 (19) Å	θ = 1.0–29.8°
*b* = 9.3282 (7) Å	µ = 3.98 mm^−^^1^
*c* = 14.1338 (19) Å	*T* = 223 K
β = 114.918 (10)°	Block, colorless
*V* = 1781.7 (4) Å^3^	0.28 × 0.26 × 0.25 mm
*Z* = 4	

### Data collection

**Table:**

Stoe IPDS 2 diffractometer	2302 reflections with *I* > 2σ(*I*)
Radiation source: fine-focus sealed tube	*R*_int_ = 0.064
graphite	θ_max_ = 29.8°, θ_min_ = 2.7°
ω–scans	*h* = −20→20
14472 measured reflections	*k* = −13→11
2544 independent reflections	*l* = −19→17

### Refinement

**Table:**

Refinement on *F*^2^	Primary atom site location: structure-invariant direct methods
Least-squares matrix: full	Secondary atom site location: difference Fourier map
*R*[*F*^2^ > 2σ(*F*^2^)] = 0.036	Hydrogen site location: inferred from neighbouring sites
*wR*(*F*^2^) = 0.087	All H-atom parameters refined
*S* = 1.12	*w* = 1/[σ^2^(*F*_o_^2^) + (0.0378*P*)^2^ + 5.0597*P*] where *P* = (*F*_o_^2^ + 2*F*_c_^2^)/3
2544 reflections	(Δ/σ)_max_ < 0.001
121 parameters	Δρ_max_ = 1.14 e Å^−^^3^
0 restraints	Δρ_min_ = −0.88 e Å^−^^3^

### Fractional atomic coordinates and isotropic or equivalent isotropic displacement parameters (Å^2^)

**Table:**

	*x*	*y*	*z*	*U*_iso_*/*U*_eq_	
Sr	0.5000	0.24637 (4)	0.2500	0.01746 (10)	
Na1	0.2500	0.2500	0.0000	0.0260 (3)	
Na2	0.5000	0.6875 (3)	0.2500	0.0440 (5)	
P	0.20406 (5)	0.76351 (7)	0.04496 (5)	0.01570 (13)	
S1	0.07286 (5)	0.67170 (8)	−0.04401 (6)	0.02445 (15)	
S2	0.18992 (5)	0.97603 (7)	0.06433 (5)	0.01959 (14)	
S3	0.28146 (5)	0.66742 (8)	0.18434 (5)	0.02452 (15)	
O1	0.59794 (17)	0.0300 (3)	0.2305 (2)	0.0318 (5)	
O2	0.50855 (17)	0.4687 (2)	0.14363 (17)	0.0259 (4)	
O3	0.41245 (15)	0.1665 (2)	0.05340 (16)	0.0238 (4)	
O4	0.31300 (14)	0.3293 (2)	0.17560 (15)	0.0220 (4)	
H1A	0.627 (5)	0.042 (7)	0.197 (5)	0.08 (2)*	
H1B	0.638 (4)	0.000 (6)	0.292 (5)	0.065 (17)*	
H2A	0.555 (5)	0.473 (6)	0.127 (5)	0.070 (17)*	
H2B	0.461 (4)	0.486 (5)	0.093 (4)	0.046 (13)*	
H3A	0.404 (4)	0.086 (7)	0.048 (5)	0.067 (17)*	
H3B	0.450 (5)	0.195 (8)	0.023 (5)	0.09 (2)*	
H4A	0.282 (3)	0.283 (5)	0.214 (4)	0.037 (11)*	
H4B	0.302 (4)	0.425 (7)	0.170 (4)	0.059 (15)*	

### Atomic displacement parameters (Å^2^)

**Table:**

	*U*^11^	*U*^22^	*U*^33^	*U*^12^	*U*^13^	*U*^23^
Sr	0.01698 (15)	0.01714 (16)	0.01694 (15)	0.000	0.00585 (11)	0.000
Na1	0.0230 (7)	0.0303 (9)	0.0221 (7)	0.0003 (6)	0.0068 (6)	−0.0014 (6)
Na2	0.0384 (10)	0.0598 (15)	0.0284 (9)	0.000	0.0086 (8)	0.000
P	0.0192 (3)	0.0137 (3)	0.0171 (3)	−0.0002 (2)	0.0104 (2)	−0.0003 (2)
S1	0.0209 (3)	0.0200 (3)	0.0336 (3)	−0.0042 (2)	0.0127 (3)	−0.0043 (3)
S2	0.0232 (3)	0.0145 (3)	0.0219 (3)	0.0012 (2)	0.0103 (2)	−0.0008 (2)
S3	0.0343 (3)	0.0226 (3)	0.0197 (3)	0.0094 (3)	0.0144 (3)	0.0058 (2)
O1	0.0232 (10)	0.0306 (12)	0.0366 (12)	0.0049 (8)	0.0076 (9)	−0.0042 (10)
O2	0.0237 (9)	0.0299 (11)	0.0224 (9)	0.0016 (8)	0.0081 (8)	0.0062 (8)
O3	0.0277 (10)	0.0218 (10)	0.0236 (9)	−0.0005 (8)	0.0126 (8)	−0.0037 (8)
O4	0.0242 (9)	0.0205 (9)	0.0229 (9)	0.0013 (7)	0.0115 (7)	0.0007 (8)

### Geometric parameters (Å, °)

**Table:**

Sr—O1	2.573 (2)	Na1—S2^iv^	2.9741 (7)
Sr—O1^i^	2.573 (2)	Na2—O2	2.570 (3)
Sr—O2^i^	2.596 (2)	Na2—O2^i^	2.570 (3)
Sr—O2	2.596 (2)	Na2—S1^iv^	2.9525 (15)
Sr—O3	2.631 (2)	Na2—S1^v^	2.9525 (15)
Sr—O3^i^	2.631 (2)	Na2—S3	2.9924 (9)
Sr—O4	2.6459 (19)	Na2—S3^i^	2.9925 (9)
Sr—O4^i^	2.6459 (19)	P—S1	2.0162 (9)
Na1—O3^ii^	2.344 (2)	P—S2	2.0243 (9)
Na1—O3	2.345 (2)	P—S3	2.0248 (9)
Na1—O4	2.372 (2)	P—P^iv^	2.2405 (12)
Na1—O4^ii^	2.372 (2)	S1—Na2^iv^	2.9525 (15)
Na1—S2^iii^	2.9741 (7)	S2—Na1^vi^	2.9741 (7)
			
O1—Sr—O1^i^	76.65 (12)	O3—Na1—S2^iii^	91.09 (6)
O1—Sr—O2^i^	149.01 (7)	O4—Na1—S2^iii^	89.18 (5)
O1^i^—Sr—O2^i^	113.34 (9)	O4^ii^—Na1—S2^iii^	90.82 (5)
O1—Sr—O2	113.34 (9)	O3^ii^—Na1—S2^iv^	91.09 (6)
O1^i^—Sr—O2	149.01 (7)	O3—Na1—S2^iv^	88.91 (6)
O2^i^—Sr—O2	73.96 (10)	O4—Na1—S2^iv^	90.82 (5)
O1—Sr—O3	73.49 (8)	O4^ii^—Na1—S2^iv^	89.18 (5)
O1^i^—Sr—O3	80.77 (8)	S2^iii^—Na1—S2^iv^	180.0
O2^i^—Sr—O3	135.60 (7)	O2—Na2—O2^i^	74.82 (12)
O2—Sr—O3	74.82 (7)	O2—Na2—S1^iv^	82.23 (5)
O1—Sr—O3^i^	80.78 (8)	O2^i^—Na2—S1^iv^	147.35 (8)
O1^i^—Sr—O3^i^	73.50 (8)	O2—Na2—S1^v^	147.35 (8)
O2^i^—Sr—O3^i^	74.82 (7)	O2^i^—Na2—S1^v^	82.23 (5)
O2—Sr—O3^i^	135.59 (7)	S1^iv^—Na2—S1^v^	127.17 (10)
O3—Sr—O3^i^	147.09 (10)	O2—Na2—S3	94.85 (7)
O1—Sr—O4	137.47 (7)	O2^i^—Na2—S3	79.40 (6)
O1^i^—Sr—O4	73.86 (7)	S1^iv^—Na2—S3	79.75 (3)
O2^i^—Sr—O4	72.30 (7)	S1^v^—Na2—S3	103.51 (3)
O2—Sr—O4	80.60 (7)	O2—Na2—S3^i^	79.40 (6)
O3—Sr—O4	72.10 (6)	O2^i^—Na2—S3^i^	94.85 (7)
O3^i^—Sr—O4	118.24 (6)	S1^iv^—Na2—S3^i^	103.51 (3)
O1—Sr—O4^i^	73.86 (7)	S1^v^—Na2—S3^i^	79.75 (3)
O1^i^—Sr—O4^i^	137.47 (7)	S3—Na2—S3^i^	172.83 (11)
O2^i^—Sr—O4^i^	80.60 (7)	S1—P—S2	111.81 (4)
O2—Sr—O4^i^	72.30 (7)	S1—P—S3	115.08 (4)
O3—Sr—O4^i^	118.23 (6)	S2—P—S3	110.55 (4)
O3^i^—Sr—O4^i^	72.10 (6)	S1—P—P^iv^	105.20 (5)
O4—Sr—O4^i^	145.98 (9)	S2—P—P^iv^	108.07 (5)
O3^ii^—Na1—O3	180.0	S3—P—P^iv^	105.57 (5)
O3^ii^—Na1—O4	97.64 (7)	P—S1—Na2^iv^	106.45 (5)
O3—Na1—O4	82.36 (7)	P—S2—Na1^vi^	137.58 (3)
O3^ii^—Na1—O4^ii^	82.36 (7)	P—S3—Na2	111.92 (4)
O3—Na1—O4^ii^	97.64 (7)	Na2—O2—Sr	105.61 (9)
O4—Na1—O4^ii^	180.0	Na1—O3—Sr	103.36 (8)
O3^ii^—Na1—S2^iii^	88.91 (6)	Na1—O4—Sr	102.17 (7)

Symmetry codes: (i) −*x*+1, *y*, −*z*+1/2; (ii) −*x*+1/2, −*y*+1/2, −*z*; (iii) *x*, *y*−1, *z*; (iv) −*x*+1/2, −*y*+3/2, −*z*; (v) *x*+1/2, −*y*+3/2, *z*+1/2; (vi) *x*, *y*+1, *z*.

### Hydrogen-bond geometry (Å, °)

**Table:**

*D*—H···*A*	*D*—H	H···*A*	*D*···*A*	*D*—H···*A*
O1—H1A···S3^vii^	0.76 (7)	2.66 (6)	3.324 (3)	146 (6)
O1—H1B···S2^viii^	0.86 (6)	2.53 (6)	3.306 (3)	151 (5)
O2—H2A···S2^vii^	0.82 (6)	2.51 (6)	3.334 (2)	176 (6)
O2—H2B···S2^iv^	0.79 (5)	2.43 (5)	3.214 (2)	176 (5)
O3—H3A···S1^ii^	0.76 (7)	2.44 (7)	3.169 (2)	163 (5)
O3—H3B···S1^vii^	0.88 (7)	2.40 (7)	3.222 (2)	157 (6)
O4—H4A···S3^ix^	0.95 (5)	2.29 (5)	3.245 (2)	175 (4)
O4—H4B···S3	0.91 (6)	2.30 (6)	3.199 (2)	171 (5)

Symmetry codes: (vii) *x*+1/2, *y*−1/2, *z*; (viii) −*x*+1, *y*−1, −*z*+1/2; (iv) −*x*+1/2, −*y*+3/2, −*z*; (ii) −*x*+1/2, −*y*+1/2, −*z*; (ix) −*x*+1/2, *y*−1/2, −*z*+1/2.

## References

[bb1] Brandenburg, K. (2004). *DIAMOND* Crystal Impact GbR, Bonn, Germany.

[bb3] Ehrhardt, C. & Gjikaj, M. (2010). *Acta Cryst.* E**66**, i54. 10.1107/S1600536810025304 PMC3007360 21588077

[bb2] Fincher, T., LeBret, G. & Cleary, D. A. (1998). *J. Solid State Chem.* **141**, 274–281.

[bb4] Jörgens, S., Mewis, A., Hoffmann, R.-D., Pöttgen, R. & Mosel, B. D. (2003). *Z. Anorg. Allg. Chem.* **629**, 429–433.

[bb5] Klingen, W., Ott, R. & Hahn, H. (1973). *Z. Anorg. Allg. Chem.* **396**, 271–278.

[bb6] Sheldrick, G. M. (2008). *Acta Cryst.* A**64**, 112–122. 10.1107/S0108767307043930 18156677

[bb7] Stoe & Cie (2008). *X-AREA* Stoe & Cie, Darmstadt, Germany.

